# Development and Application of a Multiplex qPCR Rapid Detection System for Syndromic Detection of Tick-Borne Pathogen Coinfections

**DOI:** 10.3390/microorganisms14071522

**Published:** 2026-07-12

**Authors:** Bo Yi, Ming-Qiu Fan, Si-Yi Zhao, Shi-Bo Li, Jian Chen, Hong-Xia Liu, Yong-Feng Fu

**Affiliations:** 1 Department of Medical Microbiology and Parasitology, School of Basic Medical Sciences, Fudan University, Shanghai 200032, China; 23211020213@m.fudan.edu.cn; 2 Department of Epidemiology, School of Public Health, Fudan University, Shanghai 200032, China; 3 Shanghai Municipal Centre for Disease Control and Prevention, Shanghai 201107, China; fanmingqiu@scdc.sh.cn (M.-Q.F.); chenjian@scdc.sh.cn (J.C.); 4 School of Health Sciences, University of Manchester, Manchester M13 9PL, UK; siyi.zhao-2@student.manchester.ac.uk; 5 Department of Infectious Diseases, Zhoushan Hospital, Wenzhou Medical, Zhoushan 316000, China; lsb0398@126.com

**Keywords:** tick-borne pathogen, coinfection, multiplex quantitative polymerase chain reaction, diagnosis

## Abstract

Polymicrobial coinfections are increasingly becoming prevalent within *Ixodidae* tick vectors, which considerably complicates the global burden of tick-borne diseases. This reality necessitates rapid and accurate detection methodologies capable of concurrent pathogen detection. We engineered a multiplex quantitative polymerase chain reaction (qPCR) assay targeting Spotted Fever Group Rickettsiae (SFGR), *Babesia microti*, severe fever with thrombocytopenia syndrome virus, and *Anaplasma phagocytophilum*. Performance was evaluated on plasmid standard samples and 2050 field-collected ticks, benchmarking against established conventional PCR assay. The assay achieved 10 copies sensitivity, confirmed by probit regression (limit of detection_95_: 12.4–15 copies/µL) and robust quantification (R^2^ = 0.975–0.988, *E* = 95.630–101.668%). Receiver operating characteristic analysis suggested excellent preliminary accuracy (area under the curve: 1.000; sensitivity: 100.00%; specificity: 98.57–100.00% at a pre-specified Ct cutoff of 35). Clinical validation showed high concordance with conventional PCR for single pathogens (κ = 0.977–1.000; *p* < 0.001) and 100% agreement in quadruplex coinfection detection within the limited positive cases. Therefore, multiplex qPCR technology emerges as a sensitive, rapid, extremely specific, and efficient method for real-time syndromic surveillance of tick-borne polymicrobial infections, which enhances detection in regions burdened by overlapping vectors and interconnected zoonoses.

## 1. Introduction

The accelerating convergence of climate disruption, habitat fragmentation, and arthropod expansion has catalyzed a silent revolution in the ecology of tick-borne diseases [1,2]. Pathogens once constrained by discrete enzootic cycles now increasingly occupy overlapping transmission networks across temperate biomes, which creates synergistic syndemics that defy conventional detection paradigms [3,4]. Four high-consequence agents—Spotted Fever Group Rickettsiae (SFGR), *Babesia microti*, severe fever with thrombocytopenia syndrome virus (SFTSV; Dabie bandavirus), and *Anaplasma phagocytophilum*—epitomize this epidemiological transformation, exploiting shared *Haemaphysalis longicornis* and *Ixodes persulcatus* vectors across China’s northern agricultural forest ecotones [5,6].

Beyond their expanding epidemiology, these pathogens pose direct threats to human health. SFGR (obligate intracellular, Gram-negative bacteria), *B. microti* (a blood-borne parasitic protozoan), SFTSV (Dabie bandavirus), and *A. phagocytophilum* (a strictly intracellular, Gram-negative bacterium of the *Anaplasmataceae* family) cause severe zoonoses: spotted fever rickettsioses, human babesiosis, severe fever with thrombocytopenia syndrome (characterized by thrombocytopenia and high case fatality rate), and human granulocytic anaplasmosis (involving infection of neutrophils and monocytes), respectively [7,8,9].

In addition, effective management of these high-risk tick-borne pathogens is hindered by specific, pathogen-level detection deficiencies: SFGR exhibit high tick infection rates (notably in Xinjiang) [10] yet face considerable testing limitations, such as extensive serological cross-reactivity among *Rickettsia* species and the inadequate sensitivity of conventional PCR for detecting characteristically low-level bacteremia during early acute phases [11]; SFTSV demonstrates confirmed tick-borne transmission risks in Jiangsu and Zhejiang, with evidence suggesting spillover potential—particularly concerning given the lack of rapidly deployable detection tools capable of capturing fluctuating RNA viral loads in resource-limited settings [12,13,14]; *B. microti* infections are geographically widespread, with chronicity often evading detection because traditional microscopic blood smears lack sensitivity for low-level parasitemia, and serology cannot reliably differentiate active from past infections [15]; *A. phagocytophilum* emerges as a notable zoonosis with remarkable regional endemicity, yet its molecular detection is complicated by high genetic diversity and the circulation of *A. phagocytophilum*-like variants; consequently, conventional assays frequently suffer from target mismatches and risk pathogen omission in co-infections without supplementary assays, which compromises detection accuracy [2,16].

Although several multiplex qPCR platforms have been developed, they often sacrifice either target coverage, sensitivity, or single-tube capability. A recently introduced highly effective assay [17] demonstrates excellent sensitivity (limit of detection 1 copy/μL) and specificity for the simultaneous detection of SFGR, SFTSV, and Hantaan virus. However, it omits critical sympatric tick-borne targets like *Babesia* and *Anaplasma*, which cause overlapping syndemics in East Asia. Other multiplex TaqMan assays restricted to detecting bacteria and protozoa still exhibit constraints in analytical sensitivity, with LODs of 51.41 and 49.42 copies/reaction for *Rickettsia helvetica* and *B. microti*, respectively [18]. A single-tube assay for tick screening has also been described, but it relies on SYBR Green, which is less specific than TaqMan probes and has poorer sensitivity (e.g., 100 copies for *Rickettsia*) [19]. Consequently, an integrated, single-tube platform capable of simultaneously achieving high analytical sensitivity and comprehensive cross-kingdom detection is therefore needed.

Therefore, we developed a highly sensitive multiplex quantitative PCR (qPCR) assay that simultaneously quantifies SFGR, *B. microti*, SFTSV, and *A. phagocytophilum* in ticks. This integrated approach overcomes limitations in detecting co-infections and low-diffusion pathogens, advancing syndromic surveillance, and enabling effective public health responses to tick-borne diseases.

## 2. Materials and Methods

### 2.1. Tick Collection

From March 2023 to November 2024, we obtained 2050 free-living *Ixodidae* ticks from East China (specifically across urban and rural ecotones in Shanghai and Zhejiang), representing endemic regions with known tick-borne disease transmission. All specimens were sampled from local vegetation and ground fields using the standard flagging method. Collected ticks were subjected to rigorous species determination and were confirmed as 1500 adult *Haemaphysalis longicornis* and 500 adult *Haemaphysalis flava* through both morphological examination and molecular analysis. These ticks were processed into 205 specimen pools, with each pool comprising 10 individuals. We also received nucleic acid preparations from 10 tick samples [20] (kindly provided by China CDC) that were confirmed positive for the following nontarget pathogens to establish ecological context for specificity validation: *Borrelia burgdorferi* sensu lato, *Francisella tularensis*, *Ehrlichia chaffeensis*, *Babesia divergens*, tick-borne encephalitis virus (TBEV), Powassan virus (POWV), Colorado tick fever virus (CTFV), Omsk hemorrhagic fever virus (OHFV), *Theileria orientalis*, and Alongshan virus (ALSV).

Processed ticks were then returned to storage at −80 °C until required for nucleic acid extraction.

### 2.2. Tick Processing and Genomic DNA/RNA Extraction

Prior to nucleic acid extraction, the tick specimen pools were mechanically homogenized using grinding beads. Total DNA/RNA was extracted from each specimen pool using the QIAamp Power Viral DNA/RNA Kit (Qiagen, Shanghai, China) in accordance with the manufacturer’s instructions. Extracted DNA/RNA was eluted in 50 µL nuclease-free water and stored at −80 °C.

### 2.3. Primer and Probe Design

The target genes *gltA* (for SFGR), *cox1* (for *B. microti*), *NP* (for SFTSV), and *MSP2* (for *A. phagocytophilum*) were selected to maximize analytical sensitivity and species-level specificity, guided by their known genomic characteristics and previous reports [19,20,21,22,23,24]. Conserved regions within these genes were identified via multiple sequence alignments using the ClustalW algorithm implemented in BioEdit (version 7.0.9.0), and these conserved stretches were subsequently used as templates for the design of specific primers and probes. Furthermore, all oligonucleotides, including primers for multiplex qPCR and nested PCR (Table 1 and Table 2, respectively), were designed using Oligo 7 Version: 7.60 Primer Analysis software (Molecular Biology Insights, Colorado, CO, USA). The specificity of each primer and probe set was rigorously validated through BLAST (https://blast.ncbi.nlm.nih.gov/Blast.cgi accessed on 5 May 2026) analysis against the National Center for Biotechnology Information database. Synthesis of primers and probes was performed by Sangon Biotech (Shanghai, China). The optimal oligonucleotide combinations, comprising forward primers (F), reverse primers (R), and hydrolysis probes (P), were selected along with their respective sequences, fluorophore-quencher pairs, and amplicon size.

### 2.4. Nucleic Acid Processing and Reverse Transcription

Total nucleic acid extracts, containing genomic DNA and RNA of the target pathogens (SFGR, *B. microti*, SFTSV, and *A. phagocytophilum*), were processed as follows: For RNA target (SFTSV) and DNA targets (SFGR, *B. microti*, and *A. phagocytophilum*), complementary DNA (cDNA) was synthesized using the PrimeScript™ IV 1st strand cDNA Synthesis Mix kit following the manufacturer’s protocol.

### 2.5. Preparation of Pathogen Standard Controls

The target gene fragments were amplified from cDNA/DNA of the four pathogens with specific primer pairs (Table 1) using TaKaRa Taq™ (Takara Bio, Shanghai, China), in accordance with the manufacturer’s protocol. The resulting amplicons were ligated into the pMD19-T vector using the pMD19-T Vector Cloning Kit (Takara Bio, Shanghai, China). Ligation products were then transformed into competent Escherichia coli cells. Recombinant plasmids were isolated, verified by DNA sequencing, and subsequently linearized.

Quantification of the purified plasmid DNA standards was performed using a NanoDrop™ ND-2000c Spectrophotometer (Thermo Fisher Scientific, Wilmington, DE, USA).

### 2.6. Nested PCR Detection

To establish a reliable baseline for performance comparison, we utilized nested PCR assays as reference standards. The primers for these reference assays were independently designed targeting conserved genomic regions, and the specific nested PCR protocol has been routinely optimized, validated, and employed in our laboratory for precise tick-borne pathogen identification (Table 2).

For conventional PCR, 1 µL DNA extract was prepared in a 25 µL final reaction volume, containing 0.625 U TaKaRa Ex Taq, 2.5 µL 10 × PCR buffer, 0.2 mM dNTP Mixture, and 0.4 µM outer forward and outer reverse primers (Table 2) from TaKaRa Taq™ (Takara Bio, Shanghai, China). The thermal cycle was programmed as follows: initial denaturation at 98 °C for 1 min, 35 cycles of denaturation at 98 °C for 10 s, annealing at 56 °C for 30 s, and extension at 72 °C for 1 min. The final extension was performed at 72 °C for 10 min. PCR amplicons were resolved on 2.0% agarose gels, followed by purification and sequencing. Negative samples were further analyzed by nested PCR using the primary amplicons as template under identical reaction conditions with inner primers (Table 2). Positive amplicons were sequenced and aligned against representative GenBank reference strains to validate target conservation, with detailed alignment statistics provided in Supplementary Table S1.

### 2.7. Multiplex qPCR Detection

Multiplex qPCR assays were performed in a 20.0 µL reaction volume using TaKaRa Probe qPCR Mix MultiPlus. Reaction conditions were optimized through systematic testing of annealing temperatures (54–60 °C), primer concentrations (200–2000 nM), and hydrolysis probe concentrations (100–1000 nM). Statistical validation confirmed optimal performance at 56 °C with 400 nM primers and 200 nM probes. Final reaction mixtures contained 10.0 µL 2× TaKaRa Probe qPCR Mix (Takara Bio), each of 400 nM pathogen-specific primers for *gltA* (SFGR), *cox1* (*B. microti*), *np* (SFTSV), and *MSP2* (*A. phagocytophilum*), each of 200 nM dual-labeled hydrolysis probes for corresponding targets (Table 1), 2.0 µL template DNA, and nuclease-free water to 20.0 µL.

Amplification and fluorescence detection were conducted on a 7500 Real-Time PCR System (Applied Biosystems, Foster City, CA, USA) under the following conditions: reverse transcription at 50 °C for 2 min; initial denaturation at 95 °C for 3 min; 40 cycles of denaturation at 95 °C for 15 s and optimized combined annealing/extension at 56 °C for 30 s (fluorescence signal acquisition); final extension at 56 °C for 1 min.

### 2.8. Specificity and Sensitivity Analyses

The specificity of the multiplex PCR assay was evaluated using nucleic acids of ten nontarget pathogens. Four target pathogens were concurrently analyzed as positive controls in the same qPCR run.

To strictly evaluate the analytical sensitivity under simulated co-infection conditions, the four recombinant plasmid standards were initially combined at an equimolar ratio (1:1:1:1) to generate a synthetic mixed-infection standard. Sensitivity was determined using seven serially diluted pooled multiplex standard samples (10^6^ copies/µL to 1 copy/µL) tested with six replicates in a single experimental run. The standard curve and amplification efficiency were determined via semi-logarithmic regression of threshold cycle (Ct) versus log_10_ template concentration (GraphPad Prism 9.0, San Diego, CA, USA).

Separately, a probit regression was performed to determine the limit of detection (LOD) of the multiplex qPCR assay at 95% probability using SPSS statistics software (https://www.ibm.com/products/spss-statistics accessed on 5 May 2026) (IBM Corporation, New York, NY, USA).

### 2.9. Clinical Sample Validation

All tick specimen pools underwent parallel testing via conventional PCR and multiplex qPCR. Samples tested negative by conventional PCR were further analyzed through nested PCR. For the calculation of clinical testing sensitivity and specificity, the nested PCR assay was strictly designated as the reference standard. Detection agreement between conventional PCR and multiplex qPCR was assessed by calculating Cohen’s kappa coefficient (κ) with 95% confidence intervals (CI) and performing McNemar’s chi-square test using SPSS statistics software (IBM Corporation, New York, NY, USA).

### 2.10. Receiver Operating Characteristic (ROC) Analysis

Analytical accuracy was assessed using ROC curve analysis for four pathogens (SFGR, *A. phagocytophilum*, SFTSV, and *B. microti*) with approximately 100 clinical samples per pathogen (*n* = 98–102 per group). Performance metrics (sensitivity and specificity) were assessed at the prespecified clinical cutoff (Ct ≥ 35).

## 3. Results

### 3.1. Optimization of Multiplex qPCR Assays

Reaction conditions were optimized through systematic testing of annealing/extension temperatures (54–60 °C), primer concentrations (200–2000 nM), and hydrolysis probe concentrations (100–1000 nM). Statistical validation confirmed optimal performance at 56 °C with 400 nM primers and 200 nM probes. Final reaction mixtures contained 10.0 µL 2× TaKaRa Probe qPCR Mix (Takara Bio), each of 400 nM pathogen-specific primers, each of 200 nM dual-labeled hydrolysis probes, 2.0 µL template DNA, and nuclease-free water to 20.0 µL.

### 3.2. Multiplex qPCR Specificity

**Figure 1:**
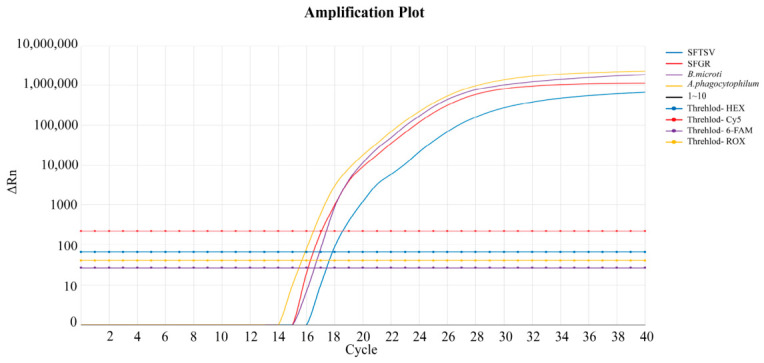
Specificity validation of the multiplex qPCR assay for detection of SFGR, *B. microti*, SFTSV, and *A. phagocytophilum*. Only the positive control well has an amplification curve; *B. burgdorferi* sensu lato, *F. tularensis*, *E. chaffeensis*, *B. divergens*, TBEV, POWV, CTFV, OHFV, *T. orientalis*, and ALSV show no reaction curves (curves 1–10).

To assess specificity of the multiplex qPCR assay, we used the nucleic acids of ten samples, which were previously confirmed positive for *B. burgdorferi*, *F. tularensis*, *E. chaffeensis*, *B. divergens*, TBEV, POWV, CTFV, OHFV, *T. orientalis*, and ALSV, respectively, as targets. None produced positive signals, and only plasmids SFGR, *B. microti*, SFTSV, and *A. phagocytophilum* yielded classical “S”-type curves, which indicates the detection method’s high specificity(Figure 1).

### 3.3. Multiplex qPCR Sensitivity

**Figure 2:**
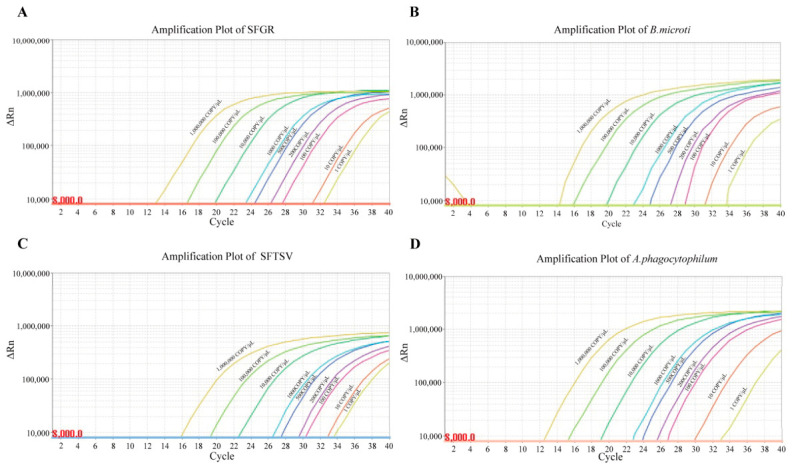
Amplification curves of the multiplex qPCR assay. Serial tenfold dilutions (10^6^ to 1 genomic copy/µL) of plasmids SFGR, *B. microti*, SFTSV, and *A. phagocytophilum* were tested. (**A**) Amplification plot of SFGR; (**B**) Amplification plot of *B. microti*; (**C**) Amplification plot of SFTSV; (**D**) Amplification plot of *A. phagocytophilum*.

**Figure 3:**
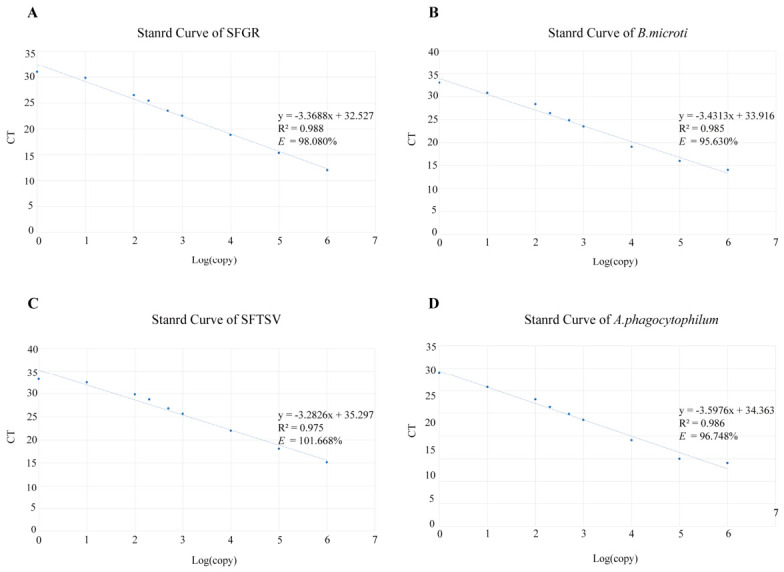
Linear standard curves and high amplification efficiencies for the multiplex qPCR assay. (**A**) Standard curve for the SFGR target (R^2^ = 0.988, *E* = 98.080%); (**B**) Standard curve for the *B. microti* target (R^2^ = 0.985, *E* = 95.630%); (**C**) Standard curve for the SFTSV target (R^2^ = 0.975, *E* = 101.668%); (**D**) Standard curve for the *A. phagocytophilum* target (R^2^ = 0.986, *E* = 96.748%). Data confirm robust quantitative performance across the measured dynamic range.

Serial tenfold dilutions of plasmid standards (10^6^ copies/µL to 1 copies/µL) were analyzed across a 7-log dynamic range (Figure 2A–D). Standard curves exhibited strong linear correlations (R^2^ = 0.988, 0.985, 0.975, 0.986) with amplification efficiencies of 98.080%, 95.630%, 101.668%, and 96.748%, respectively (Figure 3A–D), which confirmed quantitative performance across the measured range. Specifically, the regression parameters for the standard curves were as follows: SFGR (slope = −3.3688 [95% CI: −3.7001 to −3.0376], y-intercept = 32.527 [95% CI: 31.403 to 33.650); *B. microti* (slope = −3.4313 [95% CI: −3.8099 to −3.0526], y-intercept = 33.91 [95% CI:32.631 to 35.200]); SFTSV (slope = −3.2827 [95% CI: −3.7544 to −2.8109], y-intercept = 35.297, [95% CI: 33.696 to 36.897]; and *A. phagocytophilum* (slope = −3.5976[95% CI: −3.7974 to −3.4277], y-intercept = 34.363 [CI:36.574 to 32.152]). For the four pathogens, the within-run CVs ranged from 0.57% to 4.75%, whereas the within-laboratory CVs were limited to the range between 0.82% and 1.51%.

**Figure 4:**
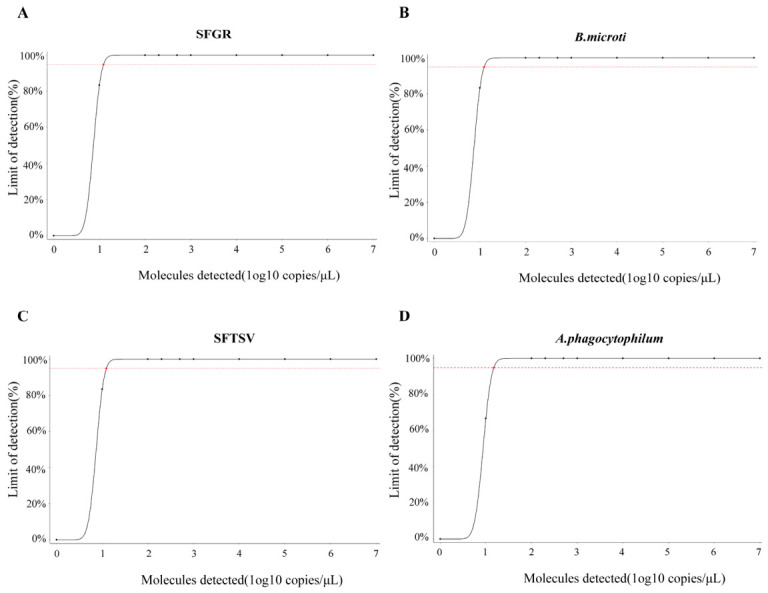
(**A**–**D**)Probit Regression Analysis of Multiplex qPCR LOD for Tick-Borne Pathogens.

Through rigorous probit regression analysis of six replicates performed on serially diluted plasmid standards, the assay’s LODs were precisely determined for all four targets. The model predicted LODs at 95% probability of 12.4 copies/µL for SFGR, SFTSV, and *A. phagocytophilum*, and 15 copies/µL for *B. microti*, demonstrating exceptional analytical sensitivity across pathogens with distinct genomic characteristics (Figure 4A–D).

Dose–response curves demonstrating the LOD at 95% probability for four pathogens: SFGR, SFTSV, *A. phagocytophilum*, and *B. microti*.

### 3.4. Multiplex qPCR Clinical Sample Validation

A total of 205 tick pools were initially screened by conventional PCR; negative pools were further tested by nested PCR, which served as the reference standard for calculating diagnostic sensitivity and specificity. All positive amplicons were verified by Sanger sequencing. Detection rates were as follows: SFGR 24.88% (51/205), *B. microti* 16.10% (33/205), SFTSV 18.54% (38/205), and *A. phagocytophilum* 26.34% (54/205).

In addition, multiplex qPCR and nested PCR were comparatively evaluated using these samples. With Ct = 35 as the clinically validated cut-off, contextual interpretation of near-threshold results within the pathogen detection continuum is critical. For single-pathogen detection, compared with nested PCR, multiplex qPCR demonstrated 100% sensitivity and 98.57–100.00% specificity. High concordance was evidenced by Cohen’s kappa coefficients of 0.977–1.000 (all *p* < 0.001) (Table 3). Co-infection panels showed 100% sensitivity and 100.00% specificity and kappa values 1.000 (all *p* < 0.001) (Table 4). Seven samples failed to produce consistent results. The multiplex qPCR-positive but conventional PCR-negative six samples were confirmed as positive by nested PCR. One false-positive result for *A. phagocytophilum* was documented in multiplex qPCR assays during comparative analysis.

### 3.5. Multiplex qPCR ROC Analysis

Multiplex qPCR exhibited superior detection discrimination (area under the curve (AUC) = 1.00) across the pathogen spectrum at Ct ≥ 35, establishing its capability for zero false-negative detections in tick specimen pools. This analytical superiority was further supported by nested PCR arbitration of seven samples with discordant results (conventional PCR-negative but multiplex qPCR-positive), of which six were confirmed positive, demonstrating high concordance with the reference method. The assay maintained operational robustness with specificity >97% against conventional PCR while detecting co-infections at near-perfect accuracy (κmax = 1.000).

## 4. Discussion

The escalating burden of tick-borne zoonoses within the Anthropocene necessitates a paradigm shift in detection surveillance. Climate-mediated arthropod expansion and anthropogenic habitat fragmentation have catalyzed the emergence of synergistic syndemics involving historically isolated pathogens [5,25]. Critically, conventional monoplex detection approaches, which are predicated on a “one-pathogen-one-test” model, are fundamentally inadequate to navigate the complex polymicrobial transmission landscapes characteristic of this era [26]. This detection void impedes accurate surveillance and obscures the true prevalence of co-infections, particularly relevant for sympatric pathogens sharing ecological reservoirs and tick vectors, a scenario acutely prevalent in economically vulnerable regions where pathogens such as SFGR (evidenced in Thai ticks with projected risk in China) [27], SFTSV (endemic in Chinese ticks) [28], *A. phagocytophilum* (posing exposure risks within China) [5], and *B. microti* (with sporadic cases and considerable importation risk in Mongolia and China) [16,29] co-circulate.

The limitations of current methods necessitate multiplex approaches. Culture isolation is impractical for surveillance due to prolonged incubation (≥72 h) and high false-negative rates, whereas monoplex PCR cannot detect co-infections. Despite enabling pathogen discovery, metagenomic sequencing is hindered by complex bioinformatics and high costs for frontline use.

Our quadruplex assay achieved amplification efficiencies of 95.6–101.7% across a dynamic range of 10^6^ to 1 copies/μL. A recently [17] reported highly sensitive multiplex assay had slightly lower efficiencies (93.5–96.9%). Some multiplex TaqMan assays can screen tick-borne pathogens comprehensively, but their efficiencies fluctuate from 95% to 114%, and they require separate reactions for different targets [18]. Their LODs are higher: 51.41 copies/reaction for *R. helvetica* and 49.42 for *B. microti*. Another single-tube multiplex assay has acceptable kinetics (90–100% efficiency) but poorer sensitivity, needing 100 copies for *Rickettsia* detection [19]. Our assay, in contrast, maintains stable kinetics and a low LOD (12.4–15 copies/μL) for all four targets in a single reaction.

This diagnostic gap risks clinical mismanagement and public health failures through undetected co-infections. Addressing this critical gap, we developed and rigorously validated a high sensitivity multiplex qPCR assay targeting these four high-priority pathogens. The assay demonstrated exclusive specificity against a broad panel of 14 taxonomically diverse tick-borne pathogens, including relevant near-neighbor agents with no cross-reactivity observed (C_T_ ≥ 35 for all nontargets; Figure 1). High analytical sensitivity was achieved. Linear quantification (R^2^ ≥ 0.975) and robust amplification efficiencies (95.630–101.668%) were maintained across an impressive seven orders of magnitude (10^6^–1 copies/µL) (Figure 2 and Figure 3). High reproducibility was confirmed via probit regression analysis, which yielded LOD_95_% values of 12.4 copies/µL for SFGR/SFTSV/*A. phagocytophilum* and 15 copies/µL for *B. microti* (Figure 4). This performance translated seamlessly to field-relevant samples: evaluation against 205 tick samples revealed near-perfect agreement with nested PCR for single infections (κ = 0.977–1.000, *p* < 0.001 (Table 3).

Our evaluation establishes multiplex qPCR could serve as a promising alternative to conventional PCR, with 100% analytical sensitivity coupled with exceptional specificity (98.57–100.00%). Multiplex qPCR achieved superior detection discrimination (AUC = 1.00) across the targeted pathogen spectrum. Crucially, six samples that yielded positive results via multiplex qPCR but negative via conventional PCR were subsequently confirmed as true positives by nested PCR. This discrepancy definitively demonstrates the inadequate analytical sensitivity of conventional PCR for low viral loads, which results in false negatives.

Despite the analytical superiority of multiplex qPCR, one false-positive result for *A. phagocytophilum* was observed against the nested PCR reference. It is important to note that this sample had a Ct value of 34.57, just below our clinical cutoff of 35. This placed it in the analytical gray zone. Given the negative nested PCR, non-reproducible duplicate signals, and lack of independent confirmation, we classified it as negative for analytical purposes; however, the possibility of a low-level true infection cannot be entirely ruled out, and this result should be interpreted as indeterminate.

Conventional PCR, however, manifested substantial detection limitations—most critically in the detection of co-infections. Two specimens confirmed as co-infected via reference methods were misclassified as negative by conventional PCR, which conclusively establishes its inadequate analytical sensitivity in poly microbial infection contexts. This observed failure corroborates with established literature on conventional PCR’s compromised efficacy in mixed-pathogen detection [26,30].

Despite its analytical strengths, this study has several limitations. The pooling approach (10 ticks/pool) meant that performance was assessed at the pool level, which does not provide individual-tick data. In addition, the findings are drawn from only two tick species, so it remains uncertain how well the assay would perform on other tick vectors. Performance metrics were derived from plasmid standards rather than field specimens. Comparative evaluations with other detection platforms (e.g., digital PCR, commercial multiplex kits, or metagenomic sequencing) would benefit from being explored in future studies. Finally, while the assay detects SFGR as a group, it does not distinguish species within this group; for studies requiring species-level resolution, complementary assays would be needed. These are important caveats, and the findings should be considered preliminary, pending further validation with larger sample sets and broader tick species.

Consequently, our multiplex qPCR assay represents an important technological advancement. It delivers high sensitivity (≤20 copies/µL), specificity, and reproducibility within a complete workflow (nucleic acid extraction to detection) requiring approximately 3 h. This condition establishes the assay as a rapid and reliable tool for active surveillance of SFGR, *B. microti*, SFTSV, and *A. phagocytophilum* in endemic regions.

## 5. Conclusions

The escalating burden of overlapping tick-borne zoonoses necessitates advanced diagnostic tools capable of identifying polymicrobial infections, overcoming the inherent limitations of conventional monoplex assays. This study successfully developed and validated a highly sensitive multiplex qPCR system for the simultaneous and rapid detection of four high-consequence tick-borne pathogens: Spotted Fever Group Rickettsiae (SFGR), *Babesia microti*, severe fever with thrombocytopenia syndrome virus (SFTSV), and *Anaplasma phagocytophilum*. The assay demonstrated exceptional analytical performance, achieving a limit of detection between 12.4 and 15 copies/µL, alongside exclusive specificity with no cross-reactivity against taxonomically diverse non-target pathogens. Ultimately, this multiplex qPCR technology provides a rapid, efficient, and highly accurate method for real-time syndromic surveillance, significantly enhancing disease monitoring and public health responses in ecotones burdened by interconnected tick-borne networks.

## Figures and Tables

**Table 1:** Primer and probe sequences for multiplex qPCR detection.

Pathogen	Gene Target	Sequence (5′ to 3′)	Probe	Product (bp)	
SFGR	*gltA*	F-TGCTCATCATTCATTAGTG	5′Cy5, 3′BHQ3	140	
		R-CTTCCTTAAAATTCAATAAATCAG			
		P-CCGACAGCCGCAAGCATAATAG			
*B. microti*	*cox1*	F-CTTCCACTTCGTCTTAAG	5′6-FAM, 3′BHQ1	246	
		R-GAACCTATACTACATAATGCA			
		P-CTTCGTCTCCGTAGTCAGGTATTCTTC			
SFTSV	*np*	F-CCTGAAGGTCGAGAATTAC	5′HEX, 3′BHQ1	196	
		R-ATCCCTGAAGGAGTTGTA			
		P-ACCTCTGTCTTGCTGGCTCC			
*A. phagocytophilum*	*MSP2*	F-GAAGGCAGTATATCCATAC	5′ROX, 3′BHQ2	178	17
		R-CTCGTAACCAATCTCAAG			
		P-CACCACCAATACCATAACCAACACTG			

Note: F, forward primer; R, reverse primer; P, hydrolysis (TaqMan) probe.

**Table 2:** Primer and probe sequences for nested PCR detection.

Pathogen	Gene Target	Sequence (5′ to 3′)	Product (bp)	
SFGR	*gltA*	Outer F-AGGAATCTTGCGGCATCGAG	594	
		Outer R-GGTCCCCAAAGTGAGGCAAT		
		Inner F-TGCTCATCATTCATTAGTG	140	
		Inner R -CTTCCTTAAAATTCAATAAATCAG		
*B. microti*	*cox1*	Outer F-TCTTAGCCTGTACTACCTCC	620	
		Outer R-ATGATAAATAGCATTGTTGAACC		
		Inner F-CTTCCACTTCGTCTTAAG	246	
		Inner R-GAACCTATACTACATAATGCA		
SFTSV	*np*	Outer F-GAGCCTTCCCACTTGGACA	333	
		Outer R-TTCAGCCACTTCACCCGAAC		
		Inner F-CCTGAAGGTCGAGAATTAC	196	
		Inner R-ATCCCTGAAGGAGTTGTA		
*A. phagocytophilum*	*MSP2*	Outer F-ATTACAGTCCAGCGTTTAGCAA	676	17
		Outer R-CCGCCTTTAAGGTCGACGTA		
		Inner F-GAAGGCAGTATATCCATAC	178	
		Inner R-CTCGTAACCAATCTCAAG		

Note: F, forward primer; R, reverse primer.

**Table 3:** Detection concordance between multiplex qPCR and nested PCR for single tick-borne pathogen detection in tick specimen pools.

TBP	Method		Nested PCR		Total	Kappa (95%CI)	*p*-Value of Kappa
			Positive	Negative			
*A. phagocytophilum*	Multiplex qPCR	Positive	32	1	33		
		Negative	0	69	69		
		Total	32	70	102	0.977(0.933–1.000)	<0.001
*B. microti*	Multiplex qPCR	Positive	30	0	30		
		Negative	0	69	69		
		Total	30	69	99	1.000	<0.001
*Rickettsia spp.*	Multiplex qPCR	Positive	31	0	31		
		Negative	0	69	69		
		Total	31	69	100	1.000	<0.001
SFTSV	Multiplex qPCR	Positive	27	0	27		
		Negative	0	68	68		
		Total	27	68	95	1.000	<0.001

**Table 4:** Analytical concordance of multiplex qPCR for polymicrobial tick-borne infection detection in tick specimen pools.

TBP	Method		Nested PCR		Total	Kappa	*p*-Value of Kappa
			Positive	Negative			
*A. phagocytophilum,* and *Rickettsia* spp.	Multiplex qPCR	Positive	8	0	8		
		Negative	0	69	69		
		Total	8	69	77	1.000	<0.001
*A. phagocytophilum, Rickettsia* spp., and SFTSV	Multiplex qPCR	Positive	9	0	9		
		Negative	0	69	69		
		Total	9	69	78	1.000	<0.001
*A. phagocytophilum, B. microti, Rickettsia* spp., and SFTSV	Multiplex qPCR	Positive	4	0	4		
		Negative	0	70	70		
		Total	4	70	94	1.000	<0.001

## Data Availability

The original contributions presented in the study are included in the article. Further inquiries can be directed to the corresponding authors.

## Supplementary Materials

The following supporting information can be downloaded at: https://www.mdpi.com/article/10.3390/microorganisms14071522/s1, Table S1: Sequence identity and BLAST analysis of detected tick-borne pathogens against reference strains.

## Abbreviations

The following abbreviations are used in this manuscript:

ALSV	Alongshan virus
AUC	Area under the curve
cDNA	Complementary DNA
CI	Confidence intervals
Ct	Threshold cycle
CTFV	Colorado tick fever virus
LOD	Limit of detection
mNGS	Metagenomic next-generation sequencing
NCBI	National Center for Biotechnology Information
OHFV	Omsk hemorrhagic fever virus
PCR	Polymerase chain reaction
POWV	Powassan virus
qPCR	Quantitative polymerase chain reaction
ROC	Receiver operating characteristic
SFGR	Spotted Fever Group Rickettsiae
SFTSV	Severe fever with thrombocytopenia syndrome virus
